# N-VEGF, the Autoregulatory Arm of VEGF-A

**DOI:** 10.3390/cells11081289

**Published:** 2022-04-11

**Authors:** Marina Katsman, Aviva Azriel, Guy Horev, Yitzhak Reizel, Ben-Zion Levi

**Affiliations:** 1 Faculty of Biotechnology and Food Engineering, Technion-Israel, Institute of Technology, Haifa 3200003, Israel; smarinap@technion.ac.il (M.K.); avival1952@gmail.com (A.A.); 2 Bioinformatics Knowledge Unit, The Lorry I. Lokey Interdisciplinary Center for Life Sciences and Engineering, Technion-Israel, Institute of Technology, Haifa 3200003, Israel; guyho@technion.ac.il

**Keywords:** VEGF-A, angiogenesis, hypoxia

## Abstract

Vascular endothelial growth factor A (VEGF-A) is a secreted protein that stimulates angiogenesis in response to hypoxia. Under hypoxic conditions, a non-canonical long isoform called *L-VEGF* is concomitantly expressed with *VEGF-A*. Once translated, L-VEGF is proteolytically cleaved to generate N-VEGF and VEGF-A. Interestingly, while VEGF-A is secreted and affects the surrounding cells, N-VEGF is mobilized to the nucleus. This suggests that N-VEGF participates in transcriptional response to hypoxia. In this study, we performed a series of complementary experiments to examine the functional role of N-VEGF. Strikingly, we found that the mere expression of N-VEGF followed by its hypoxia-independent mobilization to the nucleus was sufficient to induce key genes associated with angiogenesis, such as *Hif1α,* *VEGF-A* isoforms, as well as genes associated with cell survival under hypoxia. Complementarily, when *N-VEGF* was genetically depleted, key hypoxia-induced genes were downregulated and cells were significantly susceptible to hypoxia-mediated apoptosis. This is the first report of N-VEGF serving as an autoregulatory arm of VEGF-A. Further experiments will be needed to determine the role of N-VEGF in cancer and embryogenesis.

## 1. Introduction

**Figure 1:**
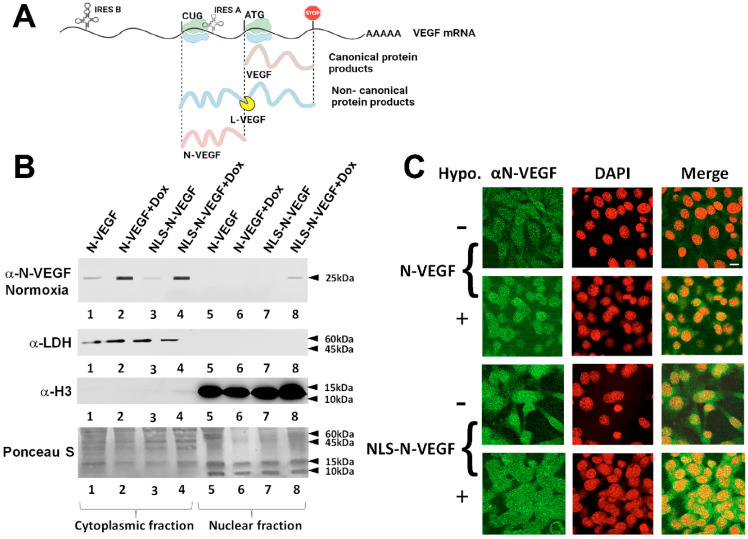
Hypoxia-independent nuclear mobilization of N-VEGF using the Cas9-NLS system. (**A**) Schematic illustration of the *VEGF* mRNA transcript, IRES domains, and the translated products. Two translational products are generated. The non-canonical *L-VEGF* translation start site is *CUG*, and the canonical start site of *VEGF* is *ATG*. The locations of IRES A and B promoting translation under hypoxic stress are indicated. VEGF and L-VEGF proteins are illustrated as well as the proteolytic cleavage site of L-VEGF resulting in N-VEGF. (**B**) NIH3T3 cells were transduced with either *N-VEGF* or *NLS-N-VEGF* expression constructs with or without the inducer of the *Tet*-on promoter (Dox). Nuclear as well as cytoplasmic fractions were obtained from these cells. Western blot with anti N-VEGF antibodies was performed on these extracts. Subsequent western blots on the same membrane were done using antibodies against LDH and histone H3 to show the purity of cytoplasmic and nuclei fractions, respectively. Ponceau S staining was carried out to demonstrate equal protein loading for all cytoplasmic and nuclear fractions. (**C**) Immunofluorescence of N-VEGF and NLS-N-VEGF cells before and following hypoxia (green) and nuclear staining with DAPI (red) is demonstrated. Minus sign represents normoxia, while plus sign hypoxia, Scale bar-50 µm.

VEGF-A (or VEGF) is a key regulator of angiogenesis in normal physiology as well as in pathological events such as cancer (for review see [1,2]). When translated under hypoxic conditions, a longer isoform of *VEGF (L-VEGF)* is generated from a non-canonical start site, CUG. L-VEGF adds 180 aa to the VEGF protein and has been detected in VEGF-producing cells, including various cancer cells [3,4,5]. Once translated, L-VEGF is subjected to a proteolytic cleavage upstream to the classical start-Met of VEGF, yielding N-VEGF and VEGF (see schematic illustration in Figure 1A). While VEGF-A is known to dramatically affect angiogenesis in surrounding cells, the functional role of N-VEGF is not known. Interestingly, ectopically expressed N-VEGF shuttles to cell nuclei only under hypoxic conditions [6]. Furthermore, immunohistochemical staining of normal angiogenic tissue samples, such as kidney and breast, demonstrated sporadic staining of N-VEGF in the nuclei of renal tubular epithelium and of epithelium cells coating the mammary ducts. Finally, renal cell carcinoma and breast cancer cell nuclei exhibited strong immunostaining of N-VEGF in all cells [6]. These observations imply the involvement of N-VEGF in transcriptional regulation of VEGF-secreting cells. However, its actual impact on gene expression and on the response to hypoxia remains a mystery.

We hypothesize that N-VEGF and VEGF play complementary regulatory roles. While VEGF is secreted and mainly affects neighboring cells, N-VEGF is retained in the cell and shuttles to the nucleus where it regulates the hypoxic transcriptional response. To characterize the role of N-VEGF in regulating transcription and in the adaptation to hypoxic stress, we performed a series of experiments. N-VEGF was mobilized to the nucleus, independent of hypoxia. Strikingly, its expression under normoxia was sufficient to upregulate key genes involved in the response to hypoxia and in the induction of angiogenesis. Complementarily, genetic depletion of *N-VEGF* inhibited the upregulation of hypoxia-induced genes and increased the apoptosis rate upon hypoxia. All these are sound proof for the key role of N-VEGF in the response to hypoxia.

## 2. Materials and Methods

### 2.1. Cell Lines

NIH3T3 (mouse embryo fibroblast) and 293FT (human embryonal kidney) were obtained from ATCC (Manassas, VA, USA) (CRL-1658 and CRL-3216, respectively). These cell lines were maintained in DMEM supplemented with 10% fetal calf serum (FCS), amphotericin (2.5 µg/mL) and gentamycin sulfate (50 µg/mL, Biological Industries, Beit-Haemek, Israel).

### 2.2. Plasmid Construction

Using PCR, a DNA fragment, encoding for *N-VEGF* (180 aa), linked to a 5′ 6× His-tag was generated, digested with the restriction enzymes EcoRI and AgeI, and then cloned into pLVX-Tet-One-Puro vector (Clontech, #631847, San Jose, CA, USA), encoding an engineered Tet activator and the corresponding doxycycline (dox)-inducible promoter. The resulting expressed protein is termed herein N-VEGF. Similarly, a DNA fragment harboring a 5′ 6× His-tag, an effective NLS, taken from the Cas9 gene, and the coding region of N-VEGF (180 aa) was generated by PCR, digested with the restriction enzymes EcoRI and AgeI, and cloned into pLVX-Tet-One-Puro. The expressed protein is herein termed NLS-N-VEGF. Clone integrity was verified by DNA sequencing.

### 2.3. Lentiviral Production

293FT cells (4 × 10^6^ cells/mL) were seeded in 10 cm tissue culture plates 24 h prior to transfection. Cells were then transfected with 5 µg lentiviral vector, 1 µg pMD.G and 4 µg psPAX2, using CalFectin, a mammalian transfection reagent (SignaGen, Frederick, MD, USA), according to the manufacturer’s instructions. The medium was changed 24 h post-transfection with 7 mL fresh medium, and 24 h thereafter, viral supernatant was collected, filtered through a 0.2 µm syringe-filter and aliquots were flash-frozen in liquid nitrogen. The viral supernatant was stored at −80 °C for further use.

### 2.4. NIH3T3 Infection

Cells (2.5 × 10^5^ cells/mL) were seeded in a 12-well tissue plate in 0.5 mL medium 24 h prior to infection. Next, 0.5 mL viral supernatant and 8 µg/mL polybrene were added to each well. The cells were incubated for 24 h at 37 °C, in a 5% CO_2_ incubator. Thereafter, the cells were split 1:2 into 6-well tissue culture plates. Stably infected cells were selected by incubation with puromycin (3 µg/mL) for 48 h and resistant cells were harvested 96 h later.

### 2.5. Nuclear and Cytoplasmic Fractionation

NIH3T3 cells (2 × 10^8^ cells/mL, 2 × 10 cm plates) were trypsinized and washed twice with ice cold PBS and lysed on ice for 15 min in a 100 µL cytoplasmic lysis buffer (10 mM HEPES pH 7.4, 10 mM KCl, 0.01 mM EDTA, 0.1 mM EGTA, 2 mM DTT, 5 mM Na_2_VO_4_, 0.1% NP40 and cOmplete, EDTA-free protease inhibitor cocktail (Sigma-Aldrich, Rehovot, Israel). Nuclei were pelleted using a benchtop microfuge (6000 rpm for 2 min) and the supernatant containing the cytoplasmic fraction was removed and treated with 2 M urea and 2% SDS and then boiled for 5 min. The nuclei were then washed twice in a 1 mL cytoplasmic lysis buffer to remove any cytoplasmic contaminant, and re-sedimented. Subsequently, the nuclei were lysed in 100 μL denaturing lysis buffer (2% SDS, 2 M urea, 8% sucrose, 1 mM NaF, and 5 mM Na_2_VO_4_). Genomic DNA was sheared by passage through a Qiashredder column (Qiagen, Crawley, UK) in a benchtop microfuge (6000 rpm for 2 min) and denatured by boiling for 5 min.

### 2.6. SDS-PAGE and Western Blotting

SDS-PAGE (15%) and western blotting were performed using a Bio-Rad Mini-protein apparatus (Hercules, CA, USA). The gel was prepared and blotted, and reacted with antibodies exactly as previously described [6]. The primary antibody used was affinity-purified rabbit polyclonal *α*ORF (N-VEGF), prepared in our laboratory against the 5’ UTRORF of VEGF (1:500 dilution in TBST + 2.5% non-fat dried milk-powder (Carnation, Markham, ON, Canada). The primary antibody was incubated for 3 h at room temperature or overnight at 4 °C. The secondary antibody, HRP anti-rabbit (1:10,000 dilution in TBST + 2.5% non-fat dried milk-powder) was incubated with the membrane for 1–2 h. Bands were visualized using an ECL2 Western Blotting kit (Thermo Scientific, Waltham, MA, USA). To demonstrate even loading of protein samples, membranes were stained, prior to membrane blocking, with Ponceau-S (Sigma-Aldrich) according to the manufacturer’s instructions and exactly as described above [7]. The purity of cytoplasmic and nuclear soluble protein extracts was determined by western blot. Membranes were stripped from anti N-VEGF or VEGF polyclonal antibodies and following re-blocking, reacted with primary antibodies directed against lactate dehydrogenase (anti-LDH) (Abcam, cat #ab47010,1:300) and secondary antibodies anti-goat IgG (1:10,000), to verify cytoplasmic extract purity. Subsequently, the membrane was stripped once more from bound antibodies, re-blocked, and reacted with antibodies directed against histone H3 acetyl K27 (αH3K27ac), primary antibody (Abcam, #ab4729, 1:500) and then with secondary antibodies, anti-rabbit IgG (1:10,000), to verify nuclear extract purity.

### 2.7. Immunofluorescence Staining and Microscopy

NIH3T3 cells, transfected with a vector driving the expression of either *N-VEGF* or NLS-*N-VEGF*, were plated in 24-well plates containing 0.13 mm microscope coverslips. Some of the samples were treated with 1 µg/mL dox for 48 h. After an additional 24 h, the cells were either not treated or subjected to hypoxia for an additional 16 h as previously described [3]. Cells were fixed with 4% paraformaldehyde for 15 min at room temperature, washed twice with PBST, permeabilized with 0.1% Triton X-100 for 15 min and washed three times. Cells were then blocked with 5% normal goat serum in TBST for 1 h and subjected to staining with primary antibodies directed against N-VEGF (1:100 dilution in blocking solution), overnight at 4 °C in a humidified chamber with very gentle swirling as described previously [6]. Finally, the coverslips were washed four times with PBS. Slides were subsequently reacted with Alexaflour 488 goat anti rabbit (H + L) (1:1000 dilution in blocking solution) for 1 h in the dark at room temperature. Following incubation, the slides were washed three times with PBS and reacted with DAPI and Fluromount-G for nuclear staining. Immunostained cells were visualized under a Zeiss LSM 700 laser scanning confocal microscope, Green laser: 555 nm (10 mW), Red laser: 639 nm (5 mW), magnification of ×30 for N-VEGF-expressing cells and magnification of ×60 for NLS-N-VEGF-expressing cells (Figure 1C). Alternatively, cells were visualized under a Zeiss Cell Observer inverted fluorescence microscope, magnification ×20 (Figure 4C).

### 2.8. Real-Time RT-PCR

The primers used for real-time PCR were designed using PrimerExpress™ 3.0 software (Applied Biosystems, Foster city, CA, USA, https://primer-express.software.informer.com/3.0/, accessed on 27 June 2019) and NCBI website tool https://www.ncbi.nlm.nih.gov/tools/primer-blast/, accessed on 27 June 2019; see Supplemental Table S1). 800 ng total RNA were reverse transcribed to cDNA using High-Capacity cDNA Reverse Transcriptase kit (Ambion, Austin, TX, USA) according to manufacturer’s instructions. cDNA was amplified with two primers for each gene using Power SYBR Green PCR Master Mix (Applied Biosystems) and Real-time PCR analysis was performed using QuantStudio 12K Flex system (Applied Biosystems, Foster City, CA, USA). The real-time PCR results were analyzed by the QuantStudio 12K Flex software version 1.2.2. https://www.thermofisher.com/il/en/home/global/forms/quantstudio-12k-flex-software-download.html (accessed on 14 July 2019). according to the manufacturer’s instructions. Only primer pairs exhibiting PCR efficiency of 90% or higher were chosen. Gene expression was normalized to GAPDH mRNA expression. The data is presented as the relative expression of the gene of interest compared with GAPDH. The mRNA expression trend of genes mentioned in this publication (up, down or unchanged) that were determined by RNA-seq, were all validated by independent real time RT-PCR.

### 2.9. RNA-Seq

RNA sample concentrations and integrity were measured using Qubit 3.0 (ThermoFisher, Wilmington, DE, USA) and TapeStation (Agilent, Santa Clara, CA, USA), respectively. Libraries for sequencing were prepared using the TruSeq RNA Library Prep Kit v2 (Illumina) according to manufacturer’s instructions. Libraries were sequenced on an Illumina NextSeq 550 instrument with 80 bps single-read. The number of reads ranged from 54,915,873 to 38,372,655 per sample. Library quality control was conducted using FASTQC, version 0.11.5, https://www.bioinformatics.babraham.ac.uk/projects/fastqc/ (accessed on 4 June 2019). Sequences were trimmed by quality using trim galore and aligned to Mus musculus genome build – GRCm38.p6 with release 94 annotation file using Tophat2, version 2.1.0 (using Bowtie2 version 2.2.6, https://sourceforge.net/projects/bowtie-bio/files/bowtie2/2.2.6/, accessed on 5 June 2019). Raw gene expression levels were counted by HTseq-count, version 0.6.1, http://www-huber.embl.de/users/anders/HTSeq/doc/count.html, (accessed on 6 June 2019). All downstream analyses were conducted in R with DESeq2, https://bioconductor.org/, (accessed on 19 June 2019) [8] as follows: raw data were filtered by independent filtering. Count normalization was performed by DESeq2 default. To identify differentially expressed genes differing between two conditions we used the Wald test, as implemented in DESeq2. Heatmaps were plotted using the R pheatmap package. For the heatmap, normalized counts were scaled by row and the genes were ordered as specified in the Figure legends. All RNA sequencing data were deposited to the Gene Expression Omnibus (GEO) with accession numbers GSE178297 and GSE178298.

### 2.10. CRISPR/Cas9-Mediated Deletion of N-VEGF 

The strategy for generating *N-VEGF* deleted cells is shown in Figure S1. sgRNAs from the 5′ and 3′ ends of the 180 aa long *N-VEGF* were designed using the chopchop online tool [9], and highly scored sgRNAs were chosen (sgRNA 1–6). Each sgRNA was phosphorylated, annealed, and cloned to lentiGuide-Puro (addgene no. 52963), which was previously digested with BsmbI. In order to create gRNAs with sticky ends complementary to BsmbI digestion product, “CACC” and “AAAC” were added to 5′ end of the designed 20-bps sgRNA in the forward and reverse oligomers, respectively. In order to verify the efficiency and specificity of the sgRNAs, a T7 endonuclease assay was performed on NIH3T3 cells and the cleavage efficiency was analyzed using TapeStation (Agilent). Two gRNAs (1 and 4) were found to specifically target the ORF: gRNA1 target-site PCR amplicon (500 bps) was cleaved to 300 and 200 bp fragments, and gRNA4 target-site PCR amplicon (461 bp) was cleaved into 329 and 132 bp fragments (data not shown). Briefly, a two-step protocol was utilized to generate NIH3T3 cells with biallelic deletion of the ORF. To get biallelic clones with reporter cassette insertion with high probability, cells were transfected with the two gRNAs and Cas9 encoding vector and two donor DNA segments (with different selectable markers, Puromycin and Hygromycin) and cultivated for two weeks. Single clones, resistant for the two antibiotics and containing the mCherry reporter gene instead of the *N-VEGF* sequence, were picked and the 5′ and 3′ junctions were assayed by PCR (Figure S2). Four Clones harboring the biallelic *N-VEGF* replacement cassette were further verified by DNA sequencing and taken for further study. For “clean” deletion of the *N-VEGF*, two clones were transduced with pMSCV-VCre encoding retroviral vector [10] to remove the reporter\selection cassettes (Figure S3). Three days later, non-fluorescent cells were harvested following two cycles of FACS sorting. *N-VEGF* deletion was verified by PCR and by DNA sequence analysis.

### 2.11. Flow Cytometry

Flow cytometry sorting was performed using a BD FACS-Aria-IIIu cell sorter (BD Bioscience, San Jose, CA, USA), and data was analyzed using Flowing Software 2 (Cell Imaging Core, Turku Centre for Biotechnology). Unstained WT NIH3T3 cells were used as a negative control and NIH3T3 cells containing mCherry reporter gene were used as a positive control.

### 2.12. Hypoxia Treatment

Hypoxic cultures were maintained in a hypoxia chamber (<0.2% O_2_) for 16 h at 37 °C in humidified 95% N_2_/5% CO_2_, and normoxic cultures were maintained in 95% air/5% CO_2_ for similar duration.

### 2.13. ApoTox-Glo^TM^ Triplex Assay

BD BioCoat Poly-D-Lysine 96-well plates with black sides and clear bottoms were used to run the ApoTox-Glo^TM^ Triplex Assay (Promega, Madison, WI, USA). Cells were trypsinized and removed from their culture flasks and diluted to 24,000 cells/mL and 100 µL of each sample was added to each well. Viability and Cytotoxicity reagents from the kit were prepared according to manufacturer’s instructions, added to a 96-well plates, and the plates was incubated at 37 °C for 1 h. After incubation, plates were analyzed for fluorescence as defined in the protocol using a Spark^®^ multimode microplate reader (Tecan, Sunrise, Switzerland) accessed on 9 December 2020. The caspase reagents were then added to the plates and the plates were incubated at RT for an additional 1 h. Luminescence was then measured. 

### 2.14. Cobalt Chloride and Tunicamycin Treatments

Cobalt chloride (Spectrum, New York, NY, USA) was added to a final concentration of 550 µM to N-VEGF NIH3T3 and the counterpart part ΔN-VEGF cell cultures. The live and dead cells were counted using trypan blue staining after 4, 6, 8 and 17 h of treatment. Next, the ApoTox-Glo^TM^ Triplex assay (Promega, Madison, WI, USA) was used to test the viability, cytotoxicity, and apoptosis of the cells as detailed above. Tunicamycin (Sigma) was added to a final concentration of 0.5 µg/mL to N-VEGF NIH3T3 and the counterpart part ΔN-VEGF cell cultures. The live and dead cells were counted using trypan blue staining after five h of treatment. Then the ApoTox-Glo^TM^Triplex assay was used to test the viability, cytotoxicity, and apoptosis of the cells.

### 2.15. Statistical Analysis

Each experiment was conducted at least three times in triplicates unless otherwise stated. Values are presented as means ± AvDev. The data was compared by unpaired two-tailed Student’s *t*-test; *p*-values < 0.05 (*) and <0.01 (**) were considered as statistically significant, as indicated in the appropriate figure. As indicated in some figure legends, two-way ANOVA was performed on the differences with cell type and various stress stimuli as the main factors. The ANOVA was followed by a post-hoc analysis of all possible contrasts using Tukey’s honestly significant difference, which controls for multiple comparisons. Contrasts with *p*-values < 0.05 are considered significant. The statistical analyses were performed using the R language and environment for statistical computing and graphics.

## 3. Results

### 3.1. Expression and Hypoxia-Independent Mobilization of N-VEGF 

To examine if the mere presence of N-VEGF in the nucleus is sufficient to induce a hypoxia-associated transcriptional response, we fused N-VEGF at its *N*-terminus to the nuclear localization sequence (NLS) of the Cas9 nuclease (NLS-N-VEGF). We chose to introduce *N-VEGF* to the nucleus under normoxic conditions to avoid the notable change that occurs in the transcriptome upon hypoxia [11]. For this purpose, *N-VEGF* and NLS-*N-VEGF* were cloned under the inducible *Tet*-on promoter in a lentiviral vector [12]. Subsequently, NIH3T3 cells expressing N-VEGF or NLS-N-VEGF were grown in the presence or absence of the Tet-on promoter inducer, doxycycline (dox). NIH3T3 cells are mouse fibroblasts cells shown in many studies to activate hypoxia-associated genes, including *VEGF-A* and *Hif1α*, upon exposure to hypoxic stress [13,14]. As seen in Figure 1B, in the presence of dox, 25% of NLS-N-VEGF was identified in the nuclear fraction. The reason for the partial nuclear localization is probably because Cas9-NLS fusion to N-VEGF resulted in reduced translocation efficiency. It is important to note that under hypoxia, only 50% of the induced N-VEGF shuttled to the nucleus (Figure 3A). Thus, only a 50% reduction in its nuclear mobilization was noted on addition of the NLS tag. Furthermore, immunofluorescence analysis confirmed that N-VEGF exhibited nuclear localization only following hypoxia (Figure 1C, second row), while NLS-N-VEGF showed nuclear expression even without hypoxia (Figure 1C, third and fourth rows).

### 3.2. Hypoxia-Independent Nuclear Mobilization of N-VEGF Induces Part of the Hypoxic Transcriptional Program

To study the effect of nuclear NLS-N-VEGF on cellular transcription, RNA was extracted from cells expressing either N-VEGF or NLS-N-VEGF and subjected to RNA-Seq. In efforts to identify altered gene expression resulting from shuttling of N-VEGF to the nucleus, while avoiding the transcriptional dynamics affected by the mere expression of N-VEGF in the cytoplasm, only genes induced in Dox-treated NLS-N-VEGF-expressing cells were considered, but neither in these cells in the absence of dox, nor in dox-treated cells expressing N-VEGF. Cells were collected after a 16-h treatment with dox to allow the translation and mobilization of N-VEGF to the nucleus. The differentially expressed genes were divided to up- and downregulated groups (fold change ≥ 2, adjusted *p*-value < 0.05) and each group was functionally annotated using Metascape [15]. 123 upregulated and 32 downregulated differentially expressed genes resulting from the shuttling of N-VEGF to the nuclei were identified (see genes list and annotations in Table S2). 

**Figure 2:**
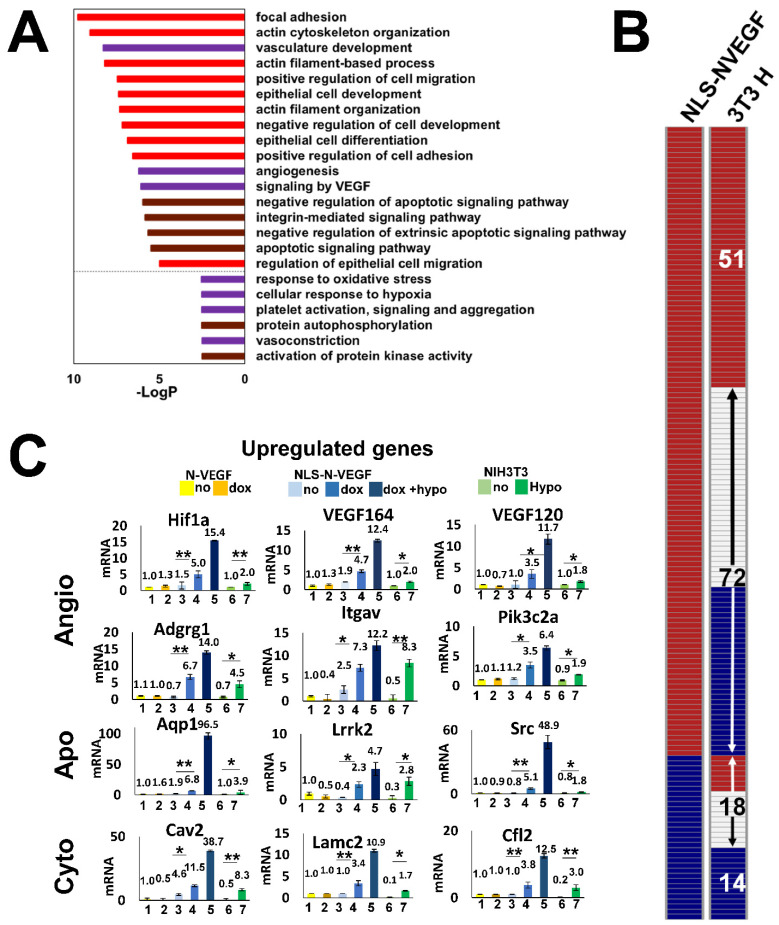
Transcriptional impact of hypoxia-independent mobilization of N-VEGF to the nucleus. (**A**) The top gene ontology terms of the 123 genes upregulated when N-VEGF is shuttled to the nucleus. Bars below the dashed line are not statistically significant. (**B**) Qualitative comparison between 155 differentially expressed genes following mobilization of N-VEGF to the nucleus and their expression trend in wild type cells exposed to hypoxia. The columns represent a qualitative heatmap showing the direction of change in gene expression level (up-red, down-blue, no change-white) in NLS-N-VEGF cells following dox treatment (NLS-N-VEGF, left column) in comparison to NIH3T3 cell under hypoxia (3T3 H, right column). The numbers within the right column represent the number of genes in each group that were annotated (detailed annotations in Figure S5A–D). (**C**) Validation of RNA seq results using qPCR. Real-time RT-qPCR analyses were performed on select upregulated genes from each functional annotation group. The data is presented for N-VEGF-expressing cells before (yellow) and following treatments with dox (dark yellow), NLS-N-VEGF cells before (light blue) and following treatments with dox (blue) and NLS-N-VEGF cells treated with dox plus hypoxia (dark blue). The data were compared to control NIH3T3 cells before (light green) and following hypoxia (green). Genes associated with angiogenesis (Angio), Apoptosis (Apo) and cell structure (Cyto) are indicated. Fold changes in mRNA levels in triplicates from three independent experiments were determined. Statistical significance was determined by two-way ANOVA, * *p* ≤ 0.05, ** *p* ≤ 0.01). Values are mean ± AVEDEV.

The annotated groups of the upregulated genes (Figure 2A) were clustered into three main categories. The first and most relevant category (Figure 2A, purple bars), represents genes promoting angiogenesis, including *Hif1α* major hypoxia-induced transcriptional regulator [16], and *VEGF* isoforms *120* and *164* (Figure 2B). Additional signature genes mediating angiogenesis, such as *Adgrg1*, *Itgav*, *Nrp*, *Flna* and *Pik3c2a*, were upregulated by mobilization of NLS-N-VEGF to the nucleus (Figure 2B, and Table S2) [17]. Another dominant annotation involved genes associated with anti-apoptotic pathways rescuing cells from hypoxic stress (Figure 2A, brown bars). Indeed, signature genes of anti-apoptotic pathways such as *Sh3rf1* [18], *Lrrk2* [19] and *Src* [20], were induced by N-VEGF shuttling (Figure 2B, and Table S2). The last and major category of annotated gene groups was associated with the integrity, structure, and maintenance of cellular cytoskeleton, which is highly relevant during hypoxic stress (Figure 2A, red bars), including *Cav2* [21], *Lamc2* [22], *Cfl2* [21], *Rock2* [23], and more (Figure 2B). Strikingly, many of the 123 upregulated genes are known targets of Hif1α, pointing to a key pathway activated by N-VEGF. Genes downregulated following NLS-N-VEGF mobilization to the nucleus included genes associated with the regulation of translation, (Figure S4). This aligns with the role of N-VEGF as an inducer of an adaptive mechanism to hypoxic crisis by inhibiting the translation machinery [24].

In order to examine whether the expression pattern of the 155 differentially expressed genes overlapped with genes induced by hypoxia, we compared NLS-N-VEGF-induced genes to hypoxia-mediated gene expression (elaborated hereafter, Figure 2B). Overall, 42% of the genes exhibited similar trends, with 51 genes induced and 14 genes downregulated in both hypoxia-induced cells and in cells showing hypoxia-independent mobilization of N-VEGF (Figure 2B). The annotated groups of upregulated genes that exhibited similar expression were grouped into the three main categories described above (Figure S5A). These observations were verified by qPCR on a set of key genes in the three groups (Figure 2C). Exposure of NLS-N-VEGF-expressing cells to both dox and hypoxia resulted in a synergistic effect, with increased expression levels of these key genes (Figure 2C, dark blue columns). Lastly, the critical role of nuclear mobilization of N-VEGF in regulating the expression of these genes was demonstrated by the lack of an effect of dox on N-VEGF-infected cells. Taken together, this focused gene profile expression analysis highlighted the role of nuclear N-VEGF in initiating part of the main hypoxic signaling cascade.

### 3.3. Genetic Deletion of N-VEGF Using CRISPR-Cas9 

**Figure 3:**
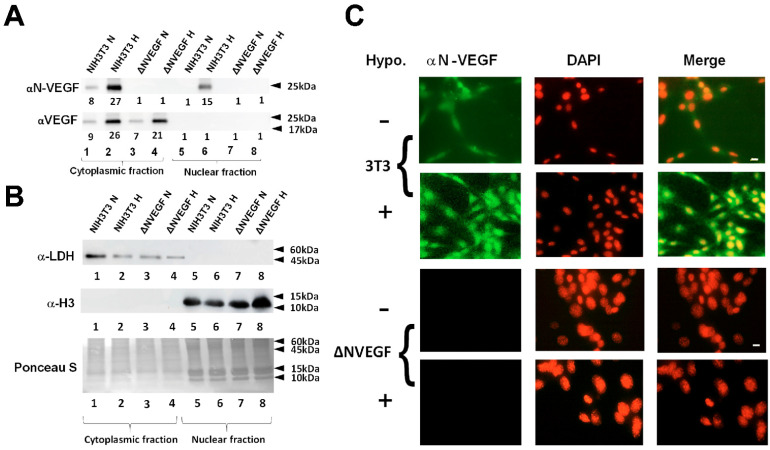
Genomic deletion of *N-VEGF* using CRISPR-Cas9. (**A**) Cytoplasmic and nuclear fractions were prepared from NIH3T3 and *N-VEGF* deleted cells (ΔNVEGF). Western blots were performed with antibodies directed against N-VEGF and VEGF showing complete deletion of endogenous *N-VEGF* using CRISPR-Cas9. Numbers below lanes indicate the average relative density of the band from three different gels as determined using ImageJ software, https://imagej.nih.gov/ij/, accessed on 12 March 2019 [25]. Statistical significance (*p* ≤ 0.05) was determined by a Student’s *t*-test. (**B**) To demonstrate the purity of soluble cytoplasmic and nuclear protein extracts, Western blots and Ponceau S staining were performed as elaborated in Figure 1. (**C**) Immunostaining showing the complete absence of N-VEGF in deleted cells was performed as elaborated in Figure 1, scale bar-50 µm.

To show the significance of endogenous *N-VEGF* expression on cellular physiology, we took advantage of the CRISPR-Cas9 genome editing system to delete only the coding region of *N-VEGF* (178 aa in mice) without affecting the translation start site of the “classical” VEGF. To measure VEGF and N-VEGF protein expression levels, we isolated soluble proteins from cytoplasmic and nuclear extracts of ΔN-VEGF cells and control NIH3T3 cells and subjected them to western blot and immunofluorescence analyses. As clearly demonstrated in Figure 3A–C, N-VEGF was entirely absent following CRISPR-Cas9 deletion. Hence, hypoxia-induced expression and nuclear mobilization of N-VEGF occurred only in wild-type cells. Importantly, N-VEGF deletion did not affect VEGF levels (Figure 3A).

### 3.4. Impact of N-VEGF Deletion on Hypoxia-Induced Genes 

**Figure 4:**
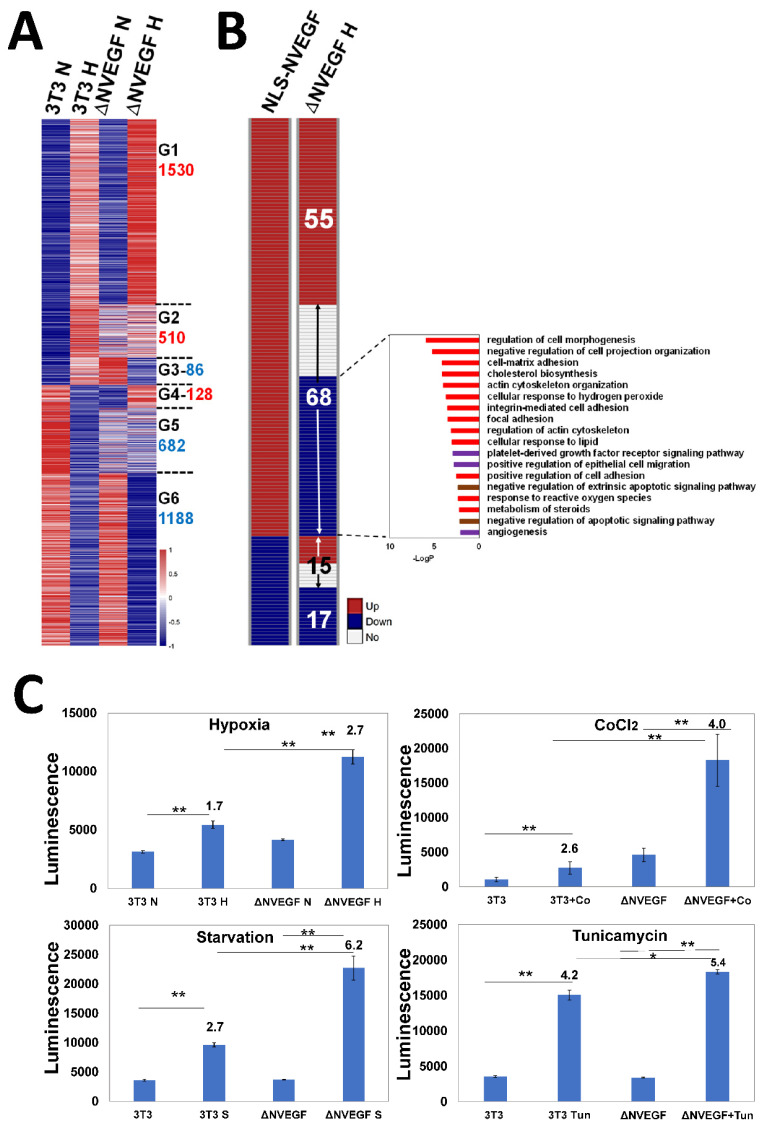
Impact of N-VEGF deletion on hypoxia-induced genes and on cellular physiology (**A**). Shown is a heatmap demonstrating gene expression levels in NIH3T3 cells (3T3) and ΔN-VEGF counterpart cells (ΔNVEGF) under normoxia (N) and following hypoxia (H). The heatmap presents only the 3000 genes modified following hypoxia in the wild type. The genes were divided into six groups (G1–G6) of upregulated (red) or downregulated (blue) expression in hypoxia. The numbers of differential genes in each group is indicated next to the name of each group (red-up and blue-down). (**B**) A qualitative comparison between the 155 genes that exhibited differential expression when N-VEGF was shuttled to the nucleus and the expression trend of these genes in ΔN-VEGF cells following hypoxia. The numbers of genes in each group is indicated in the right column. The annotations of genes (68) that were upregulated in NLS-N-VEGF cells following dox and downregulated in ΔN-VEGF cells are presented. (**C**) To induce stress signals, NIH3T3 cells and their N-VEGF-deleted counterparts were exposed to hypoxia, CoCl_2_, starvation, and tunicamycin for 18 h. Apoptosis was determined using the ApoTox-Glo^TM^ Triplex Assay. The results represent three different experiments that were performed in triplicates. Statistical significance was determined by a Student’s *t*-test, * *p* < 0.05, ** *p* < 0.01).

To show the effect of N-VEGF deletion on gene expression levels, RNA-seq analysis was performed on deleted cells and wild type cells before and following hypoxia (see Materials and Methods). A gene expression heatmap focusing on the 3000 genes modified following hypoxia in wild type versus ΔN-VEGF under the same condition is presented in Figure 4A. The genes are grouped according to expression pattern, similarity or dissimilarity, between the two cell types before or following hypoxia (G1–G6, indicated Figure 4A, and annotations in Table S3). Most genes (66%) in wild type and ΔN-VEGF cells exhibited similar expression patterns following hypoxia, as indicated in groups G1 and 6. Thus, deletion of N-VEGF led to a partial yet significant cellular response to hypoxia. Interestingly, 34% of the genes induced or repressed by hypoxia showed differential gene expression in ΔN-VEGF compared with control NIH3T3 cells (groups G2–G5). Of note, most of these genes showed differential expression in ΔN-VEGF cells even before hypoxia was induced. Following hypoxia, most of these genes showed only partial elevation or downregulation and did not reach the levels of expression observed in normal cells (groups G2 and G5). A small proportion of the genes displayed opposite expression trends under normoxia as compared to hypoxia in the two cell lines (groups G3 and G4). For example, group G3 showed higher levels of expression in the knockout compared to the wild type under normal conditions, while following hypoxia, expression levels were downregulated in the knockout and elevated in the wild type. This observation shows that N-VEGF deletion had an immense impact on gene expression even before and more massively after hypoxic stress (see annotations in Table S3). Since the main focus of this study was to characterize the nuclear role of N-VEGF, we analyzed the impact of its knockout on the 155 genes modified following N-VEGF translocation to the nucleus (Figure 4B, NLS-N-VEGF vs. ΔNVEGF). Interestingly, out of the 155 genes modified in NLS-N-VEGF cells, 53% exhibited opposite expression patterns in hypoxia-challenged ΔN-VEGF cells, which is much higher than what is expected by random events. Annotations of the genes that were upregulated by nuclear mobilization of N-VEGF and downregulated in N-VEGF-depleted cells included angiogenesis and cell survival pathways (Figure 4B), including genes associated with the negative regulation of the apoptotic signaling pathway, such as *Aqp1*, *Msn*, *Sgk1*, *Src*, *Nrp1*, and *Pik3c2a* [20,26,27,28,29,30]. This suggests that N-VEGF expression protects cells against death elicited by stress signals such as hypoxia.

### 3.5. N-VEGF Deletion Leads to Increased Hypoxia-Mediated Cell Death

**Figure 5:**
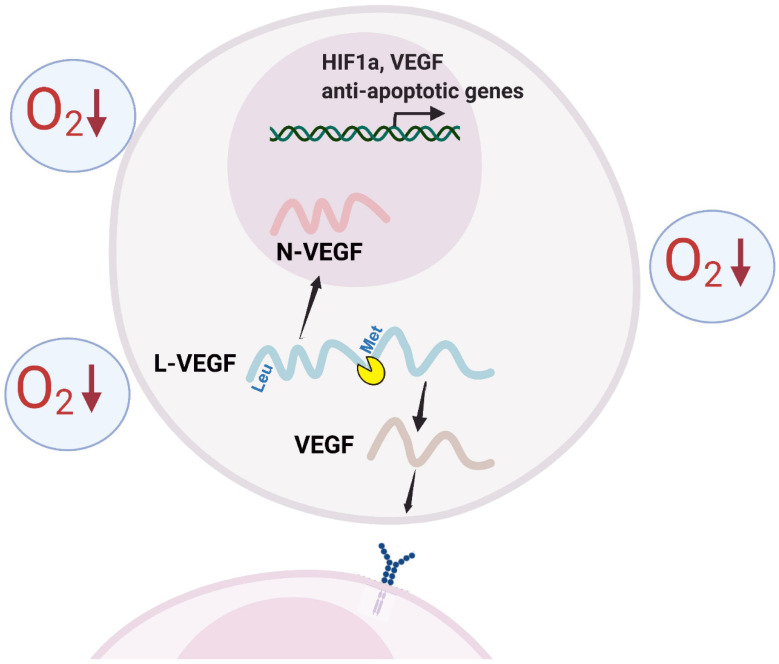
N-VEGF as an autoregulator. Schematic illustration of L-VEGF processing and autoregulation by N-VEGF. *L-VEGF* translation is initiated from an upstream non-*AUG* start codon (*CUG*-Leu). Following processing, N-VEGF shuttles to the nucleus while the remaining fragment, VEGF, is secreted via its intrinsic *N*-terminal signal peptide.

To examine if N-VEGF plays a role in defending cells against apoptosis-induced hypoxic stress, the effects of three different yet similar stress signals on ΔN-VEGF vs. wild-type cells were compared. More specifically, cells were exposed to hypoxia, CoCl_2_ [31], and serum deprivation [32], three stressors that mimic lack of blood supply. As a control, we induced Endoplasmic Reticulum stress (ER) [33], which does not mimic lack of blood supply and does not lead to VEGF expression. All stressors resembling lack of blood supply led to statistically significantly more apoptosis in ΔN-VEGF cells in comparison to NIH3T3 cells (Figure 4C, compare second and fourth columns in Hypoxia, CoCl_2_ and Starvation panels). In contrast, ER stress did not elicit a significant change in the apoptotic index between N-VEGF-deleted cells as compared to their WT counterpart cells (Figure 4C, Tunicamycin panel). Taken together, N-VEGF is essential for mediation of the anti-apoptotic state in stressed cells to overcome the harsh conditions until oxygen or blood supplies are improved. Overall, N-VEGF has a significant nuclear role on hypoxia-induced genes and physiology, as per the following proposed model (Figure 5). Upon hypoxia, the non-canonical isoform *L-VEGF* is translated and cleaved to VEGF and N-VEGF. Then, N-VEGF is mobilized to the nucleus and participates in the transcriptional regulation of the hypoxic response and in the induction of anti-apoptotic genes. While VEGF is secreted and regulates the hypoxic response in neighboring cells, N-VEGF serves as the autoregulatory arm of VEGF.

## 4. Discussion

Hypoxia is a severe cellular stress that leads to cellular injury and cell death. It leads to the upregulation of expression of gene sets that are involved in pathways aimed at minimizing cellular damage and enabling survival. Among the many rescue pathways activated in response to hypoxia are genes involved in the initiation of angiogenesis, in inhibiting apoptotic pathways, and in the maintenance of cellular structure [34]. This was demonstrated in a meta-analysis of hypoxic transcriptomes of many cell types [14]. Our data demonstrated typical changes in the transcriptome profile in the NIH3T3 cell response to hypoxia (Table S3). Likewise, nuclear localization of N-VEGF in various normal as well as cancer cell lines and tissues was also reported [3,4,5,6]. Therefore, our model of an autoregulatory role for N-VEGF (Figure 5) is physiologically relevant and represents its genuine potential.

The lack of effect of N-VEGF depletion on VEGF expression levels is explained by the existence of two separate internal ribosome entry sites (IRES) in the 5′ end of VEGF. These two IRES sequences are functional for non-canonical translation under hypoxic conditions when the normal ribosome scanning mechanism is halted (see illustration in Figure 1A) [9]. N-VEGF deletion results in impaired IRES A and IRES B is likely sufficient for effective translation of VEGF under these conditions [35,36].

This study provided complementary proof for the essential role of N-VEGF in activating the hypoxic response. We identified an exclusive gene set whose expression was modified by the mere mobilization of N-VEGF to the nucleus. Strikingly, although the hypoxic response is regulated by multiple factors, a significant number of the genes whose expression was modified when N-VEGF was shuttled to the nucleus had the inverse impact when hypoxia was induced in ΔN-VEGF cells. This set of genes included *Hif1α* and VEGF and a set of genes relevant to hypoxia. Functionally, *N-VEGF* deletion impaired the cellular defense against hypoxic stress-mediated cell death.

Although VEGF has been extensively studied in numerous physiological systems over more than three decades and N-VEGF was identified about 20 years ago, our study provides the first evidence of the functional role of N-VEGF. The regulatory architecture of the gene is comprised of two separate arms activated by the same transcript. While VEGF is secreted and activates angiogenesis in surrounding cells, N-VEGF shuttles to the nucleus and regulates intracellular gene responses, thereby serving as the autoregulatory arm of VEGF. This regulatory architecture is beneficial because it provides simultaneous regulation of both intra- and inter- cellular responses by a single promoter (see schematic illustration in Figure 5). While other biological systems, such as the pro-opiomelanocortin transcript, generate different secreted hormones by proteolytic cleavage (for review see [37]) or delta-notch signaling, in which a segment of the notch receptor is sent to the nucleus and regulates transcription (for review see [38]), to our knowledge, this is the first report of a mechanism in which the same transcript regulates intra- and inter- cellular response. It would be interesting to identify additional transcripts having similar regulatory architectures.

The extent to which N-VEGF plays a regulatory role in hypoxic responses and in angiogenesis in physiological and pathophysiological contexts remains to be determined. Circumstantial evidence suggests the involvement of N-VEGF in various pathologies. Genomic polymorphism resulting in IRES B dysfunction was shown to reduce L-VEGF levels in ALS, in macular retinal thickness, and found to be associated with an increased risk of breast cancer aggressiveness, and gastric as well as prostate cancers (reviewed in [1]). The role of N-VEGF in models of these diseases as well as in normal development remains to be examined.

Since this study uncovered a previously unknown regulatory mechanism of VEGF, many elementary questions regarding the manner in which N-VEGF exerts its effect remain to be addressed. Future experiments will determine if N-VEGF binds to the chromatin, elucidate the genomic loci it interacts with and identify its partners. In addition, deciphering its three-dimensional structure will help in understanding its specific role.

## Data Availability

All RNA sequencing data were deposited to the Gene Expression Omnibus (GEO) with accession numbers GSE178297 and GSE178298.

## Supplementary Materials

The following supporting information can be downloaded at: https://www.mdpi.com/article/10.3390/cells11081289/s1, Figure S1: Schematic illustrations; Figure S2. PCR analysis of NIH3T3 with a replacement of the *N-VEGF* gene with a reporter cassette; Figure S3:. PCR analysis to verify genomic *N-VEGF* deletion from NIH3T3; Figure S4: Annotation and pathway analysis of the 32 differentially expressed genes that were down regulated following hypoxia independent nuclear mobilization of NLS-N-VEGF; Figure S5: Annotation of 155 genes that exhibited differential expression in NLS-N-VEGF cells and NIH3T3 cells following hypoxia; Table S1: List of primers used in this study; Table S2: Gene list and annotation of 155 genes that exhibited differential expression in NLS-N-VEGF cells treated with dox; Table S3: Annotation of genes that exhibited differential expression between Δ-N-VEGF and NIH3T3 cell following hypoxia.
