# Supplementary material for: N-VEGF, the Autoregulatory Arm of VEGF-A
Source: Cells. 2022 Apr 11;11(8):1289. doi: 10.3390/cells11081289 (PMC9024919; doi:10.3390/cells11081289)
Supplement: Supplementary file 1 [file cells-11-01289-s001.zip › Supplementa Data.pdf]

Supporting information for

N-VEGF, the autoregulatory arm of VEGF-A

Marina Katsman[1], Aviva Azriel[1], Guy Horev[2], and Yitzhak Reizel[1†], and Ben-Zion Levi[1*]

*Corresponding author. Email: blevi@technion.ac.il

†Co-corresponding author, e-mail: y.reizel@technion.ac.il

This PDF file includes:

Figs. S1 to S5
Table caption S1 to S3

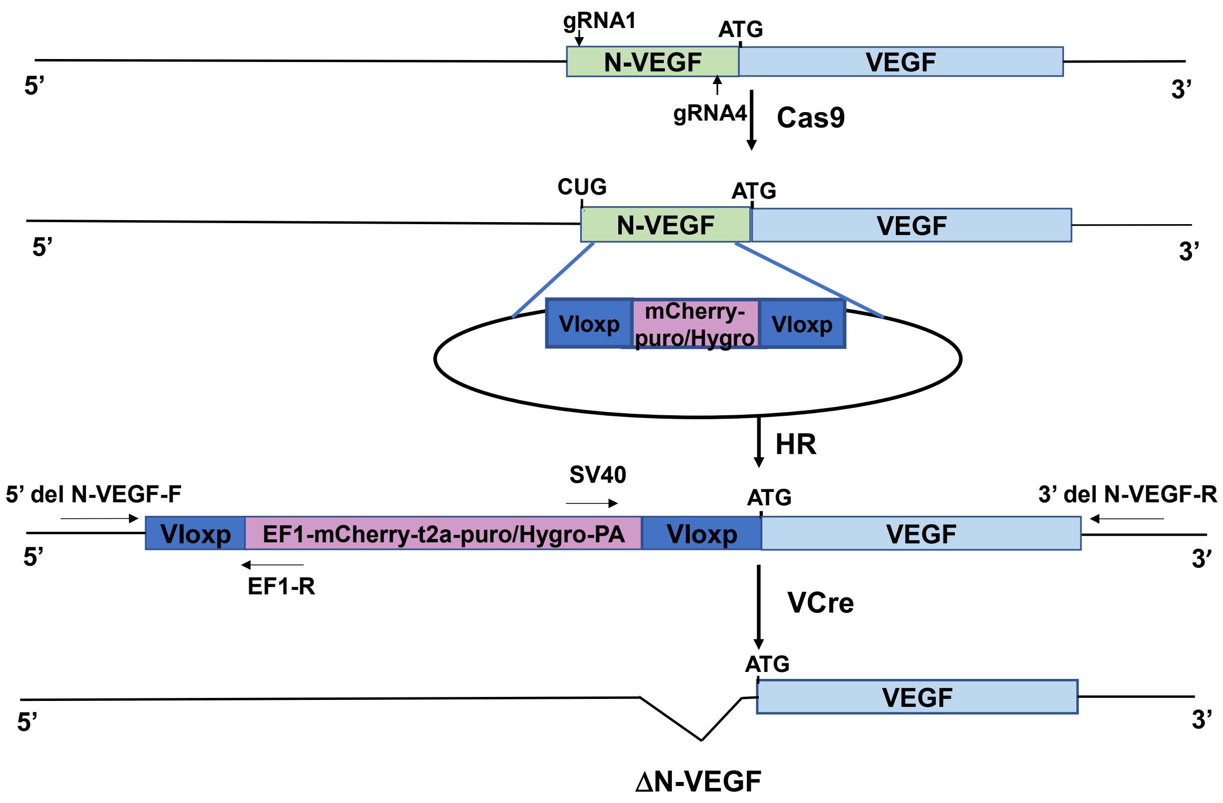

Figure S1. Schematic illustrations. Schematic illustration of the two-steps protocol for CRISPR-Cas9 mediated biallelic deletion of N-VEGF.

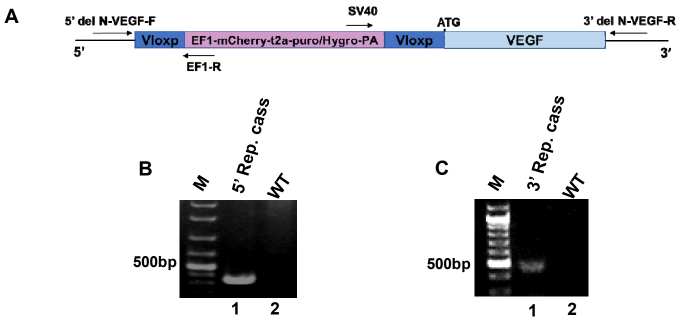

Figure S2. PCR analysis of NIH3T3 with a replacement of the N-VEGF gene with a reporter cassette. (A) Schematic illustration of the reporter cassette harboring a reporter gene followed by Puromycin or Hygromycin resistance genes. Primer pairs for each junction were used for clones analysis. **(B)** Agarose gel showing the expected 435bp PCR product from 5' junction of isolated clone 1 (panel 1) obtained using the 5'-del NVEGF forward primer and reverse primer EF1-R from the insert. **(C)** Agarose gel showing levels of the expected 482bp PCR product from the 3' junction (panel 1), obtained using the SV40 forward primer and 3' del NVEGF reverse primer.

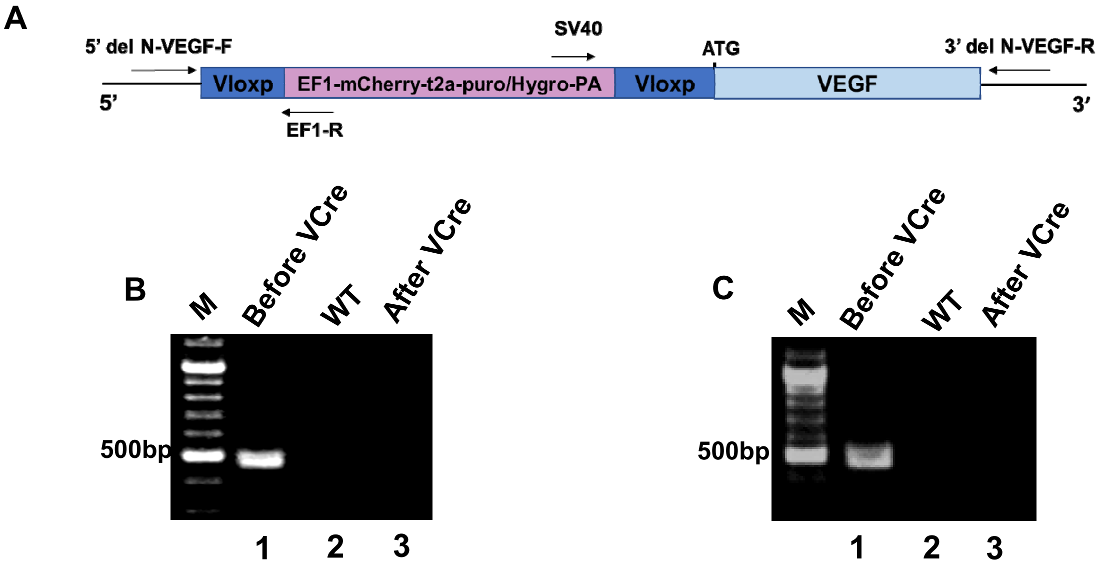

Figure S3. PCR analysis to verify genomic N-VEGF deletion from NIH3T3. (A) Schematic illustration of the reporter cassette harboring a reporter gene followed by Puromycin or Hygromycin resistance genes. Primer pairs for each junction were used for clones analysis. **(B)** Agarose gel showing levels of the expected band of 435 bp PCR product from the 5' junction of clone 1 before (panel 1) and following VCre expression (panel 3). Removal of the reporter cassette was determined using 5'-del NVEGF forward primer and reverse primer EF1-R from the insert. **(C)** Agarose gel showing levels of the expected band of 482 bp PCR product from the 3' junction, obtained using the SV40 forward primer and 3'-del NVEGF reverse primer before (panel 1) versus after VCre expression (panel 3).

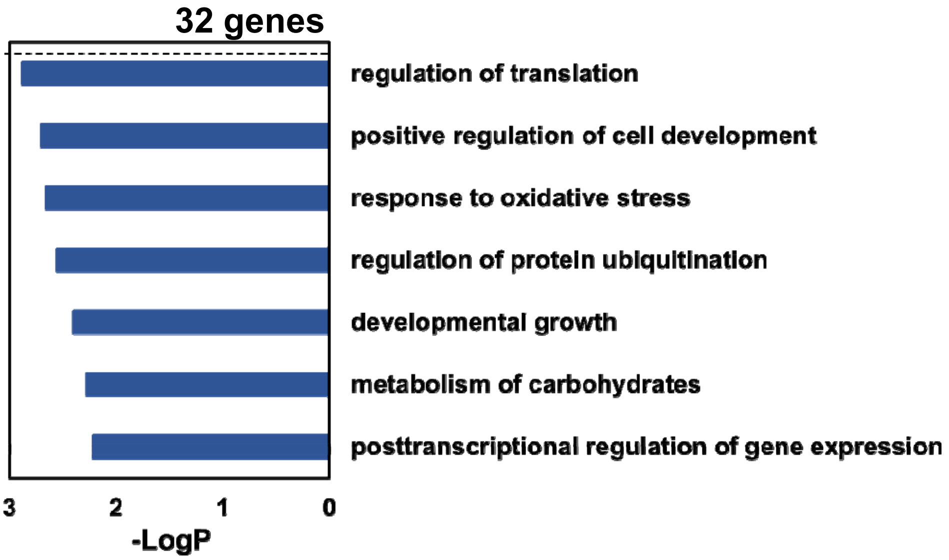

Figure S4 Annotation and pathway analysis of the 32 differentially expressed genes that were down regulated following hypoxia independent nuclear mobilization of NLS-N-VEGF. The annotations for the downregulated genes was performed as elaborated under Fig.1.

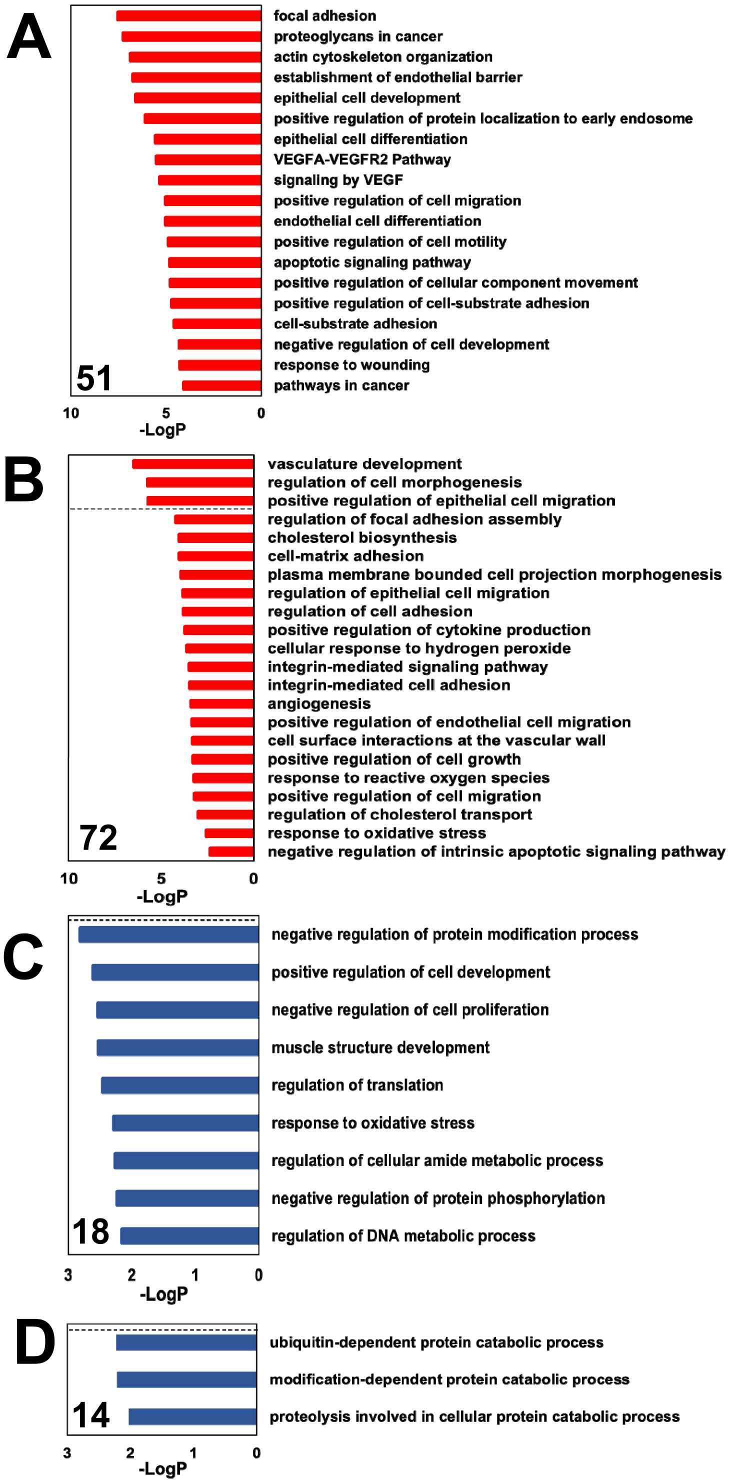

Figure S5 Annotation of 155 genes that exhibited differential expression in NLS-N-VEGF cells and NIH3T3 cells following hypoxia. (A) Annotation of genes (51) that were upregulated in both cell types. (B) annotation of genes (72) that were upregulated in NLS-N-VEGF cells following dox and not changed or downregulated in NIH3T3 cells. (C) annotation of genes (18) that were downregulated in NLS-N-VEGF cells following dox and upregulated or not changed in NIH3T3 cells. (D) Annotation of genes (14) that were downregulated in both cell types. Bars below dotted line represent less significant annotation groups.

Table S1. List of primers used in this study.

Table S1.xlsx

Table S2. Gene list and annotation of 155 genes that exhibited differential expression in NLS-N-VEGF cells treated with dox. Annotation of 123 upregulated genes (up tab) and 32 downregulated genes (down tab) in NLS-N-VEGF cells treated with dox.

Table S2.xlsx

Table S3. Annotation of genes that exhibited differential expression between Δ-N-VEGF and NIH3T3 cell following hypoxia. The genes are arranged in six groups (G1-G6). G1 and G6, similar expression pattern in both cell types of genes either induced or repressed following hypoxia, respectively. G3 and G4, inverse expression pattern in Δ-N-VEGF cells, either upregulated (G3) or downregulated (G4) following hypoxia in comparison to NIH3T3 under the same treatment. Last, genes that exhibited no change in expression pattern in ΔN-VEGF cells that were either upregulated or downregulated in NIH3T3 cells following hypoxia, groups G2 and G5, respectively. Statistically significant annotation groups, Log $q < 0.05$.

Table S3.xlsx
